# Influence of Dimethylsulfoxide and Dioxygen in the Fructose Conversion to 5-Hydroxymethylfurfural Mediated by Glycerol's Acidic Carbon

**DOI:** 10.3389/fchem.2020.00263

**Published:** 2020-04-08

**Authors:** Tatiane C. Tudino, Renan S. Nunes, Dalmo Mandelli, Wagner A. Carvalho

**Affiliations:** Center for Natural Sciences and Humanities, Federal University of ABC (UFABC), Santo André, Brazil

**Keywords:** acid-assisted hydrothermal carbonization, sulfonated carbons, glycerol, fructose dehydration, DMSO, 5-HMF

## Abstract

Both the catalytic production of 5-hydroxymethylfurfural (5-HMF) from carbohydrates and the use of a catalyst obtained from residues stand out for adding value to by-products and wastes. These processes contribute to the circular economy. In this work it was evaluated optimized conditions for 5-HMF production from fructose with high yield and selectivity. The reaction was catalyzed by an acidic carbon obtained from glycerol, a byproduct of the biodiesel industry. Special attention has been given to the use of dimethyl sulfoxide (DMSO) as a solvent and its influence on system activity, both in the presence and absence of O_2_. Glycerol's carbon with acidic properties can be effectively used as catalyst in fructose dehydration, allowed achieving conversions close to 100% with 5-HMF selectivities higher than 90%. The catalyst can be reused in consecutive batch runs. The influence of DMSO in the presence of O_2_ should be considered in the catalytic activity, as the stabilization of a reaction intermediate by the [O_2_:DMSO] complex is favored and, both fructose conversion and 5-HMF yield increase.

## Introduction

**Figure 1:**
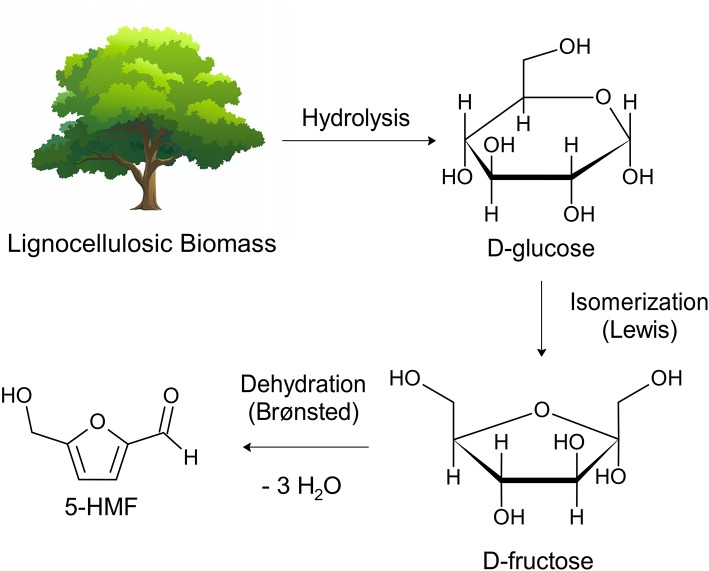
Production of 5-HMF from biomass.

5-Hydroxymethylfurfural (5-HMF) is considered as a platform molecule composed by a furan ring and aldehyde and alcohol functional groups. This structure favors its conversion into several chemical compounds with wide industrial applicability, from fine chemistry to biofuels (Putten et al., 2013). It can be obtained by dehydration of all types of C6 carbohydrates (Figure 1), including monomers and polymeric carbohydrates such as fructose, glucose, sucrose, starch, inulin and cellulose, as well as other biomass raw materials (Teong et al., 2014; Nunes et al., 2020). The use of glucose-based polysaccharides or glucose monomers as starting materials in the reaction is economically preferred if we consider that these substrates are more abundant than fructose. However, converting these types of substrates generally requires severe reaction conditions and consequently less selectivity for 5-HMF due to the decomposition and polymerization reactions of this product (Fang et al., 2014; Yi et al., 2015; Shahangi et al., 2017).

The use of heterogeneous catalysts has been studied for the production of 5-HMF due to several advantages compared to typically used homogeneous catalysts, such as the reduced reactor corrosion and reduced residue formation (Yi et al., 2015). However, solids commonly used as support for the reaction-active acidic functional groups [silicas (Wang et al., 2019), aluminas (Tong et al., 2010; Solis Maldonado et al., 2017), zeolites (Wang et al., 2018), zirconias (Ni et al., 2019), etc] typically require extensive synthesis processes. Generally acidic groups are inserted by post-synthesis processes by adding numerous preparation steps. In addition, in many cases high concentrations of functionalizing agents, such as organic and mineral acids, or high temperatures are used (Fang et al., 2014). Among the types of heterogeneous catalysts most used in carbohydrate dehydration are carbons, which can be produced from carbon-based agro-industrial residues. These processes have been highlighted by adding value to waste, contributing to the Circular Economy in the industry (Adib et al., 2015). A wide variety of wastes, including coffee grounds (Gonçalves et al., 2016b), rice husks (Boonpoke et al., 2013), palm tree (Adinata et al., 2007), coconut (Daud and Ali, 2004), almond (González et al., 2009), glycerin and glycerol (Monteiro et al., 2018), has already been used for this process and the carbons obtained have a wide applicability, being used as catalysts in many hydrogenation reactions (Madduluri et al., 2020), environmental catalysis (Restivo et al., 2017), and photocatalysis (Velasco et al., 2013). The high temperatures used in the carbonization of these residues result in catalysts with poorly developed porosity and surface area, and some carbons undergo a later stage of chemical activation to increase both their porosity and surface area (Lin et al., 2019). Although several reaction types show a significant improvement in results, it is experimentally observed in carbohydrate dehydration that a higher selectivity to 5-HMF is not dependent on a higher surface area, but rather on a higher concentration of total acidic sites on the carbons surface (Wang et al., 2018). High selectivity to 5-HMF was obtained in a previous study using glycerin and glycerol-based catalysts with a low surface area. The catalysts were obtained after only 15 min of carbonization and *in situ* functionalization (Mantovani et al., 2018). A high density of total acidic groups of 4.1 mmol/g was obtained in the catalyst. Sulfonated and oxygenated groups, such as carboxylic acids and lactones, were found on their surface (Mantovani et al., 2018). Their presence has been shown to be crucial for dehydration of fructose. Zhao et al. (2016) produced glycerol carbons with only oxygenated groups on their surface and by testing their catalytic activity in fructose dehydration, a 64.3% conversion of fructose was obtained with a 5-HMF selectivity of 57%. The introduction of sulfonic groups led to fructose conversion reaching 97% and 5-HMF selectivity of 77%. However, a loss in 5-HMF selectivity was also observed when using carbon with high number of sulfonic groups.

Although the interaction between fructose and sulfonic groups is predominant, higher densities of total acid groups have shown a greater influence on 5-HMF yield and selectivity compared to higher densities of strong acidic groups. The synergistic action of sulfonic and oxygenated groups allows a better catalytic performance for the production of 5-HMF (Thapa et al., 2017). Recently, Yu et al. (2020) also demonstrated that Brønsted acid sites with adjacent carboxyl present higher catalytic ability than isolated ones.

The search for green solvents has been carried out by several researchers, but without success so far. Ideally, water should be used as a solvent. However, in the presence of acidic species 5-HMF tends to easily decompose upon rehydration, forming levulinic and formic acids as byproducts (Wang et al., 2019). Therefore the production of 5-HMF in aqueous systems occurs parallel to a high yield of levulinic and formic acids (Yi et al., 2015).

The yields obtained in aqueous media are much lower, essentially when compared to those obtained using DMSO, a solvent widely used in the production of 5-HMF (Wang et al., 2011, 2019). DMSO-based solvent systems are one of the most attractive due to DMSO abundance, low cost, manufacturing by an environmentally friendly process, low toxicity, and the outstanding properties when compared to competing solvents and potential applications. Currently some studies have been performed in order to analyze the influence of DMSO on 5-HMF production and an apparent catalytic activity has been proposed by decomposition in H_2_SO_4_ (Tong and Li, 2010; Zhang et al., 2016), or by solvation (Kimura et al., 2013; Whitaker et al., 2019). Amarasekara et al. (2008) found a change in the anomeric composition of D-fructose in DMSO when the solution is heated to 150°C, with subsequent formation of 5-HMF after 15 min reaction time. Some examples of DMSO activity in fructose dehydration are found in the literature (Musau and Munavu, 1987; Amarasekara et al., 2008; Shimizu et al., 2009; Binder et al., 2010; Liu et al., 2014; Hu et al., 2015). In these works, 5-HMF yields range from 1.8 to 72% at temperatures in the range of 100° to 160°C. Typically, the highest yields were obtained at higher temperatures and in the presence of O_2_. However, there is no assessment of the influence of O_2_ on reaction systems. Thus, the objective of this work is to evaluate the best conditions to obtain higher selectivities to 5-HMF using carbon as a catalyst as well as to analyze the influence of DMSO as solvent in this process.

## Materials and Methods

### Chemicals

Carbon was produced utilizing glycerol 99% from Vetec (São Paulo, São Paulo), sulfuric acid >98% from LabSynth (Diadema, São Paulo) and acetone from Cosmoquimica Indústria e Comércio (Barueri, São Paulo). Characterization techniques used KBr 99.5% from Sigma-Aldrich (São Paulo, São Paulo), NaOH 99% from Vetec (São Paulo, São Paulo), NaHCO_3_ 99.5% from Sigma-Aldrich (São Paulo, São Paulo), HCl 36.5–38.0% from Vetec (São Paulo, São Paulo). Catalytic tests were performed with D(-)fructose >99,9% from Sigma Aldrich (São Paulo, São Paulo) and Dimethylsulfoxide >99,9% from Synth (Diadema, São Paulo). All chemicals were used as received and without any treatment.

### Catalyst Preparation

Carbon was prepared as reported in a previous study (Mantovani et al., 2018). In a typical process, glycerol (10 g) was mixed with concentrated sulfuric acid (30 g) in a Teflon-coated stainless-steel autoclave reactor. Then the system was heated to 180°C in an oven and kept to that temperature for 15 min. At the end of the process the solid obtained was washed repeatedly with distilled water until a pH value above 4 and then with acetone to remove non-carbonized organic matter. Precipitation tests with BaCl_2_ were also performed to confirm the absence of sulfate ions in the washing solution. The obtained materials were oven dried at 60°C for 6 h. The washing process was repeated twice under the same conditions.

### Catalyst Characterization

Surface functional groups were analyzed by Fourier Transform Infrared spectroscopy (FTIR) analysis using a Varian-Agilent 640-IR FT-IR Spectrometer. The analyses were performed mixing dried carbons with KBr in a 3:100 weight ratio and ground into fine powder. These mixtures were dried at 60°C for 12 h and thin pellets were made in a manual press. The spectra were acquired by accumulating 32 scans at 4 cm^−1^ resolution in the 4,000–400 cm^−1^ range.

X-ray photoelectron spectroscopy (XPS) data were obtained on a K-alpha + ThermoFisher Scientific or a VG-Microtech Multilab 3000 spectrometer, equipped with a hemispherical electron analyzer with X-ray source Mg Kα (*h* = 1253.6 eV) 300 W X-ray source.

Boehm's method (Boehm, 1994) was used to estimate the acid-base properties of samples For the test, 300 mg of the carbon was added in 25 mL of NaOH 0.1 mol/L to quantify all Brønsted acids groups in the carbon surface or NaHCO_3_ 0.1 mol/L to quantify sulfonic and carboxylic acid groups. A blank was prepared for each experimental condition. The suspensions were degassed with N_2_, and the bottles were closed and shaken for 24 h at room temperature. Aliquots of filtered solutions were titrated with HCl 0.1 mol/L in an automatic titrator Metrohm 905 Titrando.

Carbon, oxygen, and sulfur content were obtained in an elemental analyzer (EA1112 Thermo Scientific FLASH) using 3 mg of samples previously dried at 60°C for 12 h.

### Catalytic Tests

Reactions were performed in a Teflon-lined stainless-steel reactor (Parr 4848). In a typical run, a 2.5 or 5 wt.% fructose solution in DMSO was added in the reactor with 2.5–10 wt.% of catalyst and kept under mechanical stirring for 2 h under synthetic air or N_2_. The temperature was kept at the desired value, ranging from 80 to 130°C. Blank reactions (in absence of catalyst) were also performed.

The reaction products were analyzed by high performance liquid chromatography (HPLC Agilent 1200 series, equipped with a refractive index detector and ROA Organic Acid column, 300 × 7.8 mm). The mobile phase used was a 4 mmol/L aqueous solution of H_2_SO_4_ previously filtered through 0.45 μm membrane and degassed by ultrasound. The column was maintained at 60°C with 0.6 mL/min mobile phase (isocratic) flow. Reaction products were identified and quantified by pure standards and previously constructed analytical curves of fructose, 5-HMF, levulinic acid and formic acid. Fructose was detected by Refractive Index Detector (RID) at 40°C while 5-HMF, formic acid and levulinic acid were detected by Diode Array Detector (DAD) at 254, 210, and 210 nm, respectively.

The stability of the prepared materials was evaluated by performing consecutive batch runs under the same reaction conditions, in order to investigate the reusability of the catalyst. After each reaction run, the solid was removed from the reaction solution, washed with acetone to remove the reactants adsorbed on the surface and dried at 333 K for 12 h and then used for the next run without any reactivation or purification.

Fructose conversion into products and yield and selectivity for the identified products (5-HMF, levulinic acid and formic acid) were calculated as follows.

(1) $$\begin{array}{l} Conversion\;\left(\%\right)=\left(\frac{moles\;of\;fructose\;reacted}{initial\;moles\;of\;fructose\;}\right)\times 100 \end{array}$$

(2) $$\begin{array}{l} Yield_{prod}\;\left(\%\right)=\left(\frac{moles\;of\;product}{initial\;moles\;of\;fructose\;}\right)\times 100 \end{array}$$

(3) $$\begin{array}{l} {\;Selectivity}_{prod}\;\left(\%\right)=\frac{Yield_{prod}}{Conversion} \end{array}$$

## Results and Discussion

### Catalyst Characterization

**Figure 2:**
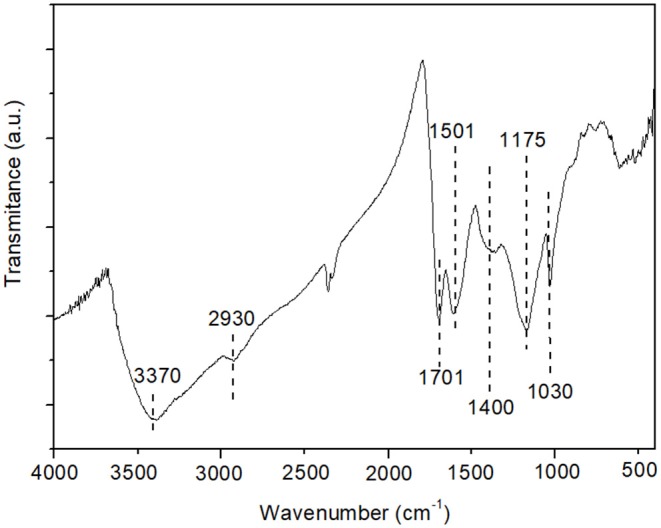
FTIR spectrum for the prepared carbon.

The methodology used in this work allowed the formation of sulfonic and carboxylic acidic groups in the carbon as can be seen by FTIR analysis (Figure 2). The presence of sulfonic acidic groups is confirmed by characteristic bands observed in the spectrum at 1,030 and 1,175 cm^−1^. These bands correspond to the asymmetric and symmetrical vibrational stretching of SO_2_, respectively (Zhao et al., 2016; Mantovani et al., 2018). The 3,370 cm^−1^ centered broadband is attributed to O-H stretching of -COOH groups while the band around 1,590 cm^−1^ can be attributed to the C=C double bond stretch characteristic of carbons (Thapa et al., 2017; Yu et al., 2020). One band at 1,701 cm^−1^ is characteristic of carbonyls from -COOH group and another at 2,930 cm^−1^ is attributed to the elongation vibrations of methylene groups (Zhao et al., 2017).

A high density of total acidic groups was obtained by Boehm Titration. The amount of total acidic groups was 4.2 mmol/g, of which 1.7 mmol/g correspond to carboxylic and sulfonic acid groups. In addition to the strong acid groups, groups with weak, and moderate acidity are also produced, including lactones, phenols and carbonyls, as previously reported (Gonçalves et al., 2016a). As expected, the non-activation of carbon and the *in situ* functionalization method used resulted in a high concentration of total acidic groups even with the use of a lower precursor: acid ratio (Table 1).

**Table 1:** Values of total acid groups in non-activated and activated carbons.

**Reference**	**Carbon**	**Precursor:acid mass ratio**	**Functionalization**	**Total acidic groups (mmol/g)**
This work	Non-activated	Glycerol: sulfuric acid (1:3)	15 min at 180°C	4.20
Gonçalves et al. (2016b)	Non-activated	Coffee grounds: sulfuric acid (5:92)	2 h at 180°C	3.00
Gonçalves et al. (2016b)	Non-activated	Coffee grounds: sulfuric acid (5:92)	5 h at 180°C	3.40
Wataniyakul et al. (2018)	Non-activated	Glycerol: sulfuric acid (5:92)	15 h at 150°C under N_2_	1.08
Mo et al. (2008)	Non-activated	D-glucose: sulfuric acid (1:36,8)	13 h at 160°C	0.60
Maneechakr and Karnjanakom (2017)	Non-activated	β-ciclodextrin: hydroxyethyl sulfonic acid (1:4)	4 h at 180°C	1.82
Lin et al. (2019)	Activated	Furfural residue: sulfuric acid (1:36,8)	5 h at 150°C	2.72
Ormsby et al. (2012)	Activated	Biochar: sulfuric acid (12,5:36,8)	12–18 h at 100°C	2.59
Villanueva and Marzialetti (2018)	Activated	Activated carbon: sulfuric acid (5:184)	16 h at 150°C	5.30

Unlike what was done in this work, the methodology of preparation of non-activated carbons developed by Gonçalves et al. (2016a) and by Wataniyakul et al. (2018) considered the sulfuric acid functionalization step after hydrothermal carbonization. Although these authors used a longer functionalization time, a higher temperature and a higher precursor: acid mass ratio, a lower density of total acidic groups was obtained compared to the results obtained in this work. *In situ* sulfonation favors the formation of a greater number of functional groups throughout the carbonaceous structure that is being produced. As the structure is less organized, it is easier to insert acidic groups. Thus, keeping the functionalizing agent present while the precursor is carbonized, it is possible to increase the amount of functional groups formed throughout the structure, whether on the surface or not. Thus, the *in situ* functionalization method allowed reaching a higher density of acidic groups on the carbon surface.

The pyrolysis carbonization method is another factor that hinders the functionalization process of the carbons. Mo et al. (2008) promoted pyrolysis of D-glucose under N_2_ for 15 h at 400°C. Then, the carbon obtained was sulfonated using precursor: acid mass ratio of 1:36.8 at 160°C for 13 h. A lower density of total acidic groups was obtained compared to the values obtained in this work. It has been suggested that carbon produced from pyrolysis processes has a higher degree of crosslinking as treatment is done at higher temperatures (Xiong et al., 2018). This is the reason why activated carbons cannot be easily functionalized (the more graphitized the structure, the more difficult it is to functionalize). The increase in temperature associated or not with a longer carbonization time, increases the fixed carbon content, while the ratios of O/C and H/C decreased. The number of acidic functional groups also decreased as a function of pyrolysis temperature, especially carboxylic groups (Marsh and Rodríguez-Reinoso, 2006; Saleh, 2011). Thus, the carbon structure becomes more hydrophobic and less flexible, making it difficult to insert acidic groups in a later stage of functionalization (Zhong and Sels, 2018). In this case, it is common to use high concentrations of acids at elevated temperatures for more efficient functionalization (Ormsby et al., 2012; Villanueva and Marzialetti, 2018; Lin et al., 2019). On the other hand, the presence of carboxyl and hydroxyl groups in sulfonated carbon can make the surface more polar for substrate adsorption such as fructose and lead to hydrogen bond formation (Dong et al., 2015). Sulfonated carbons are promising materials and can be used as acidic catalysts due to its high density of surface acidic groups, especially carboxylic and sulfonic, acting as Brønsted acid sites. However, other functional groups with acidic characteristics present on the surface (such as phenolic and lactonic) influence the catalytic activity. Xiong et al. (2018) proposed that these groups can act by establishing hydrogen bonds with the carbohydrates tested as substrate and increasing the polarity of the carbon surface, improving its contact with the sulfonic and carboxylic groups. Recently, Yu et al. (2020) showed that adjacent acid sites on carbon surface cooperatively catalyze the fructose dehydration. The adjacent Brønsted sites lead to co-interaction with the fructose, then accelerating the dehydration. The co-interaction also results in more stable dehydration intermediates, enhancing the selectivity.

From the elemental analysis of the carbon the composition 65.9% C, 3.8% H, 3.4% S, and 26.9% O was obtained. Considering the glycerol mass composition (39.1% carbon, 8.8% hydrogen, and 52.1% oxygen) there is, as expected, an increase in the carbon content and a decrease in the amount of oxygen and hydrogen due to the carbonization process. X-ray photoelectron spectroscopic analysis indicated the composition 78.2% C, 19.5% O, and 2.3% S. When comparing these values with those obtained by elemental analysis, it is noted that the O and S contents are lower than those previously obtained. This is because the XPS analysis considers only the elements present on the carbon surface and therefore available to act as active sites.

**Figure 3:**
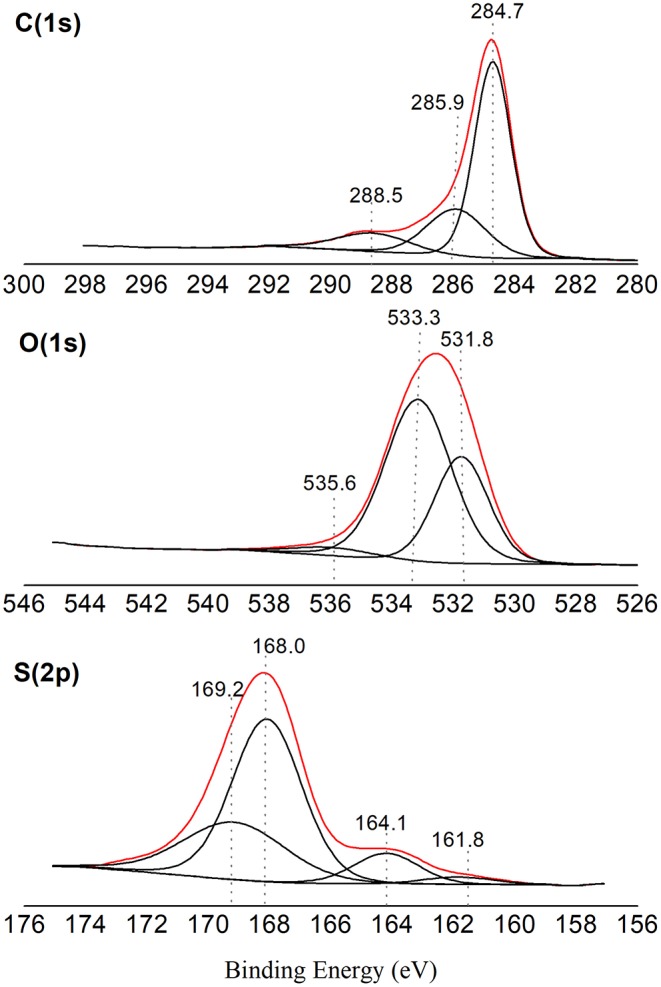
XPS spectra (red lines) for carbon, oxygen and sulfur, and their deconvolution fitting (black lines).

From the XPS analysis, it was also possible to identify the oxidation state of each element on the carbon surface, as can be observed in the spectra shown in Figure 3. The carbon spectrum (C1s) shows the presence of C–C bonds with centralized bonding energy at 284.7 eV. The presence of oxygen groups has also been confirmed by the peaks at 285.9 and 288.5 eV, which are attributed to C–C and COOH carbon bond belonging to carboxylic groups, respectively (Shimizu et al., 2009; Binder et al., 2010). In addition, peaks at 531.8 and 533.3 eV in the oxygen spectrum can also be attributed to the C–OH and C=O bonds, respectively, confirming the presence of these groups.

The sulfur spectrum (S2p) peaked at 168.0 eV is assigned to a higher oxidation state of sulfur, –SO_3_H (Aldana-Pérez et al., 2012). Peaks at 161.8 and 164.1 eV observed in the S2p spectrum belong to photoelectrons of groups with reduced sulfur forms (Fraga et al., 2016).

### Catalytic Tests

**Figure 4:**
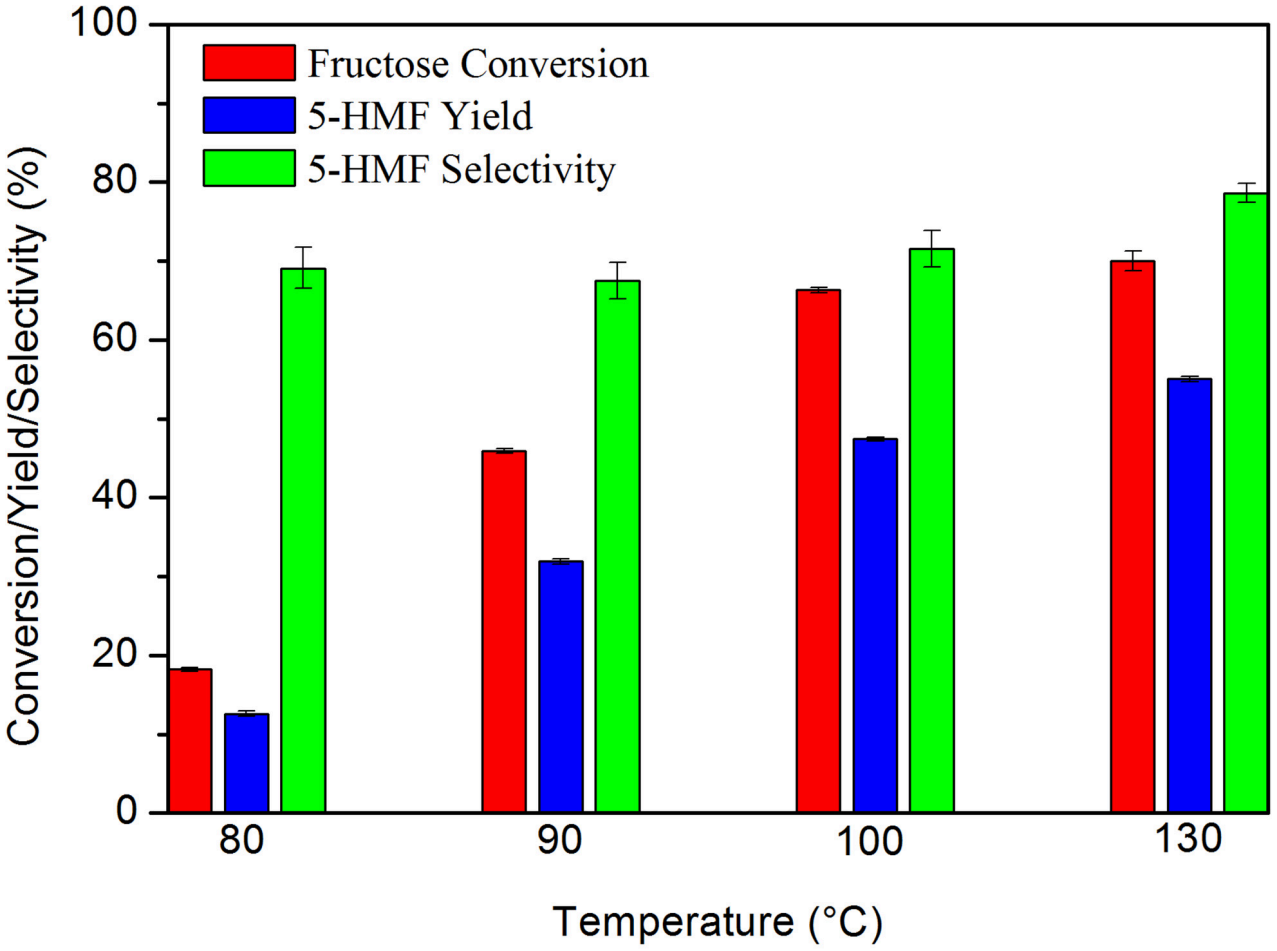
Fructose dehydration reaction at different temperatures. Conditions: 5% fructose in DMSO, 5 wt.% catalyst, 2 h reaction time under synthetic air.

Preliminary tests allowed to evaluate the behavior of the catalytic system in relation to the temperature and the carbon: substrate mass ratio. The results showed that the increase in temperature favorably influenced the process, leading to an increase in both fructose conversion and 5-HMF yield, without losses in its selectivity. By increasing the temperature from 80 to 130°C, the yield of 5-HMF increased from 12.6 to 55.6% (Figure 4).

In the literature this behavior was also observed by Zhao et al. (2016), who obtained a conversion of 12.8% and a selectivity of 40.5% at 100°C. By increasing the temperature to 160°C these values increased to 100 and 76.3%, respectively. Wang et al. (2011) also obtained an increase in 5-HMF yield by increasing the temperature from 70 to 130°C, reaching a yield of 91.2% at 130°C. However, these authors used catalyst: substrate mass ratios as high as 0.8:1 and 5-HMF selectivity decreased quickly at temperatures above or below 130°C. The authors justified this behavior due to the existence of partial dehydrated intermediates and the formation of humins, respectively.

**Figure 5:**
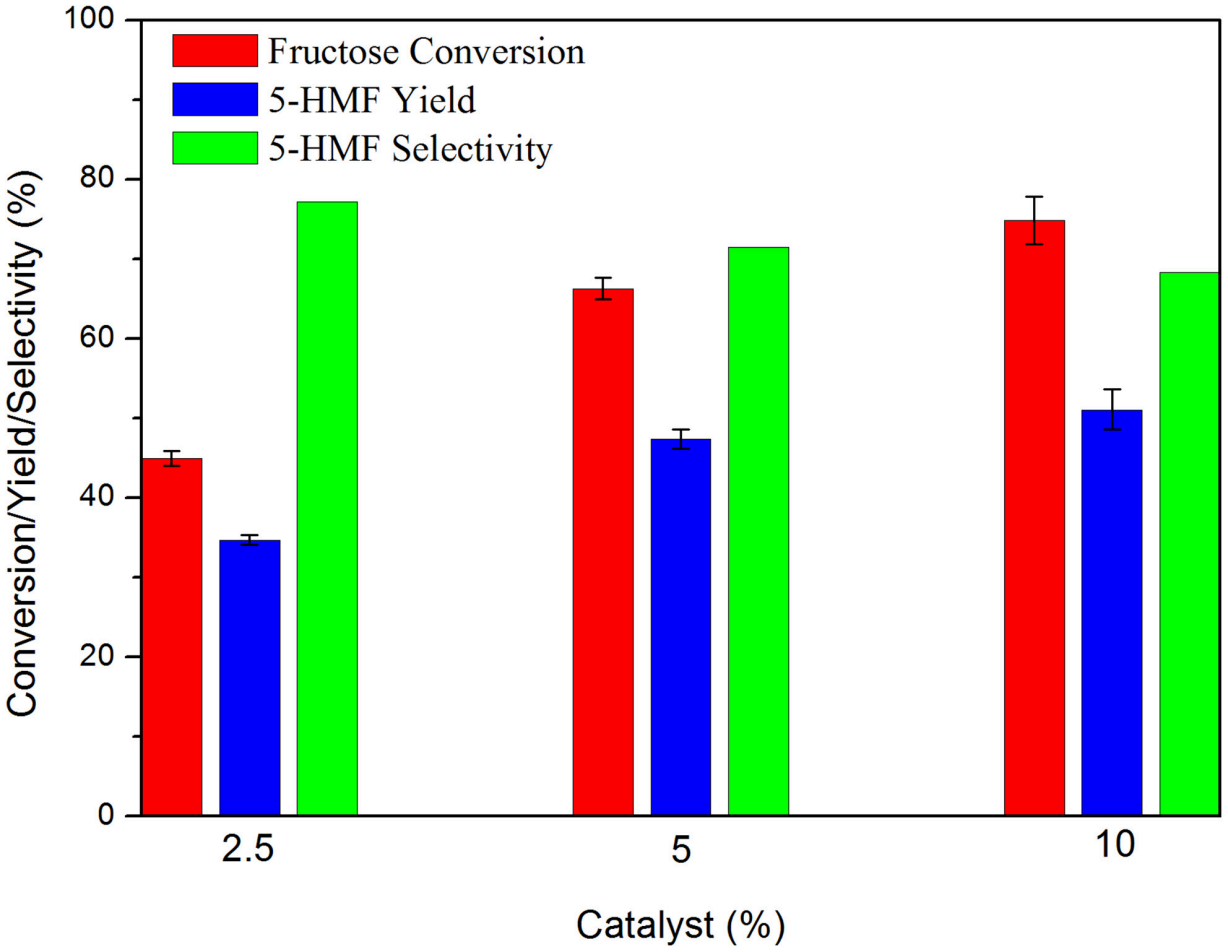
Fructose dehydration reaction in the presence of different catalyst:substrate mass ratios. Conditions: 5% fructose in DMSO, 100°C, 2 h reaction time under synthetic air.

Often, the literature brings results promoted by the use of high amounts of catalyst. In this work the best amount of carbon for the reactions was 5 wt.% relative to the substrate, which resulted in the highest yield and selectivity values for 5-HMF. The use of 10 wt.% increased fructose conversion but decreased 5-HMF selectivity resulting in minimal difference in yield (Figure 5).

The decrease in 5-HMF selectivity can be attributed to the increased number of available acidic sites due to the increased amount of carbon used. This condition favored rehydration and polymerization reactions of 5-HMF, as previously discussed. Zhao et al. (2016) were able to increase 5-HMF yield from 60.7 to 75.4% by increasing the catalyst: substrate mass ratio from 5 to 20%. However, by increasing the mass ratio to 40% 5-HMF yield decreased to 70.8%.

**Figure 6:**
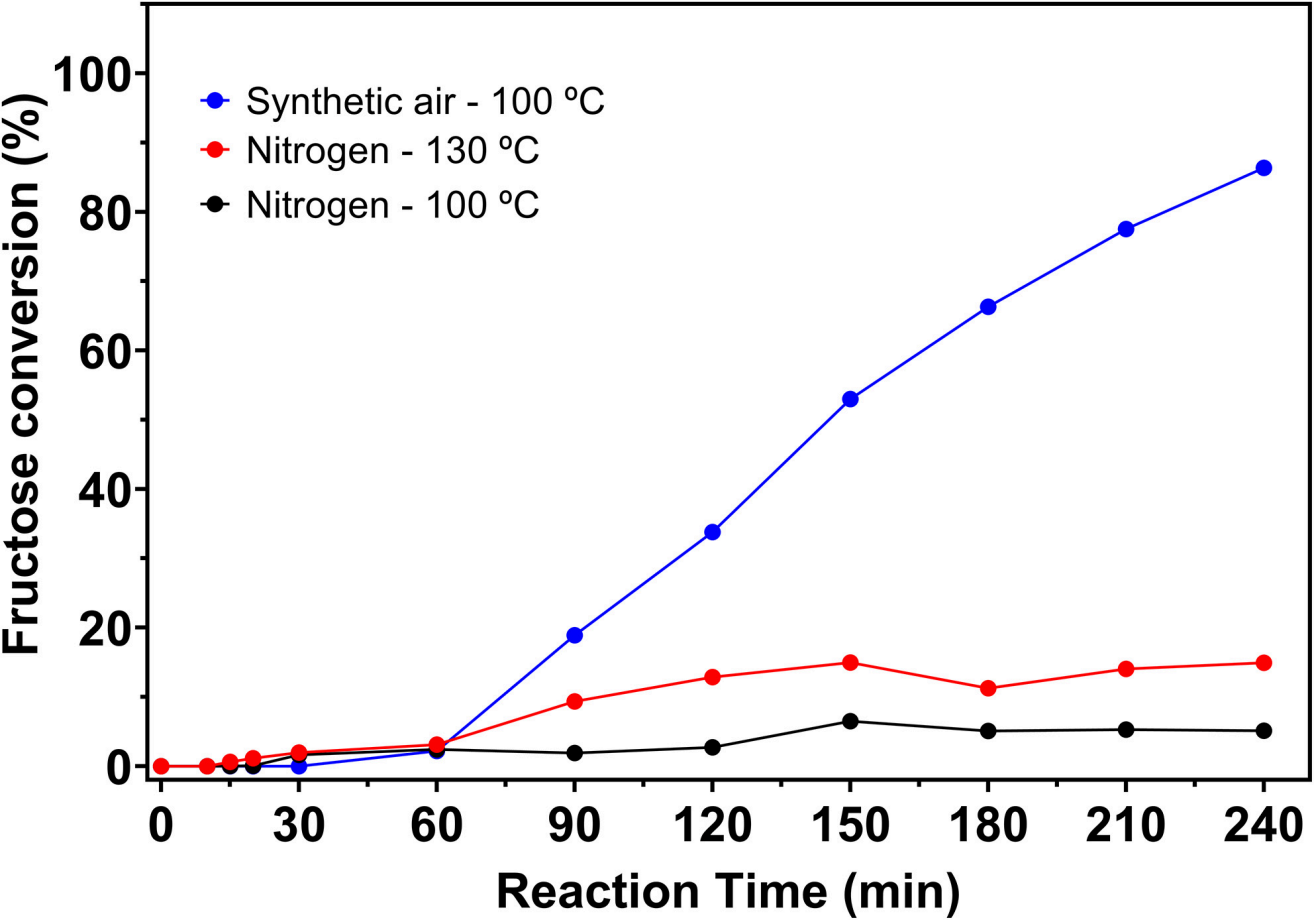
Fructose conversion values for blank reactions under nitrogen at 100 and 130°C and under synthetic air at 100°C, from 5% fructose solutions in DMSO.

**Figure 7:**
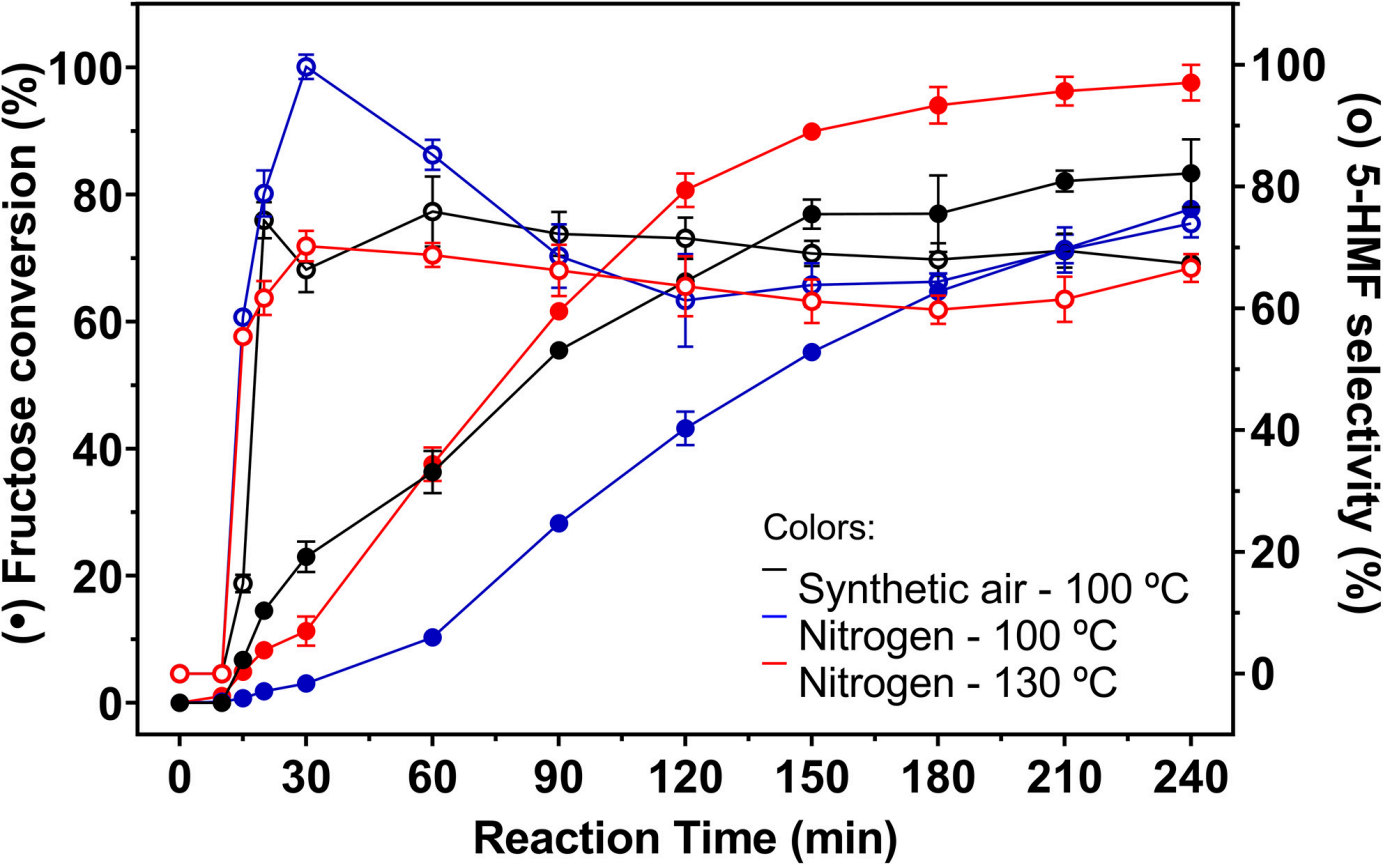
Fructose conversion and 5-HMF selectivity values (full and empty symbols, respectively) for carbon catalyzed reactions under inert atmosphere at 100 and 130°C and under synthetic air at 100°C. Conditions: 5% fructose in DMSO, 5 wt.% catalyst.

Thus, different catalytic tests were performed with solutions containing 5% fructose and 5 wt.% catalyst, varying the temperature (100 or 130°C), the reaction atmosphere (synthetic air or N_2_) and the reaction time. Catalytic tests were also performed in the absence of catalyst, whose fructose conversion values can be seen in Figure 6. Fructose conversion and 5-HMF selectivity values obtained in the carbon catalyzed reactions are shown in Figure 7.

In blank reactions, it is evident that the solvent is catalytically active for fructose dehydration in the presence of O_2_. Even under inert atmosphere, a reduced activity was observed. In all cases fructose conversion started only after 30 min of reaction. Zhang et al. (2016) demonstrated that, under inert atmosphere, DMSO stabilizes the intermediate carbocation resulting from the removal of the first water molecule from fructose, with the formation of a [carbocation-DMSO] complex. This complex is immediately converted to other intermediates as the second and third water molecules are removed from fructose, when 5-HMF is then produced. Therefore, it can be considered that the induction period observed in blank reaction is related to the time required to remove the first water molecule from fructose.

In the presence of O_2_, the system is much more active. Some authors have proposed that the catalytic activity of DMSO is attributed to its decomposition into H_2_SO_4_, which in turn is catalytically active in the reaction (Tong and Li, 2010; Zhang et al., 2016). However, recently a detailed study by Whitaker et al. (2019) concluded that DMSO does not decompose at temperatures below 190°C and the catalytic activity of the solvent would be attributed to “solvation effects.” Ren et al. (2017) when performing theoretical studies concluded that the homogeneous species [DMSOH^+^], catalytically active, can be formed during the reaction when acidic Brønsted species are present. In the absence of acidic species, DMSO stabilizes an intermediate of fructose dehydration, similar to that proposed by Zhang et al. (2016). This ability of the solvent to stabilize the reaction intermediate is responsible by the conversion, even limited, of fructose and 5-HMF production under inert atmosphere. In addition to this ability, the results of this work combined with literature data indicated the positive effect of the presence of O_2_ on both fructose conversion and 5-HMF production. Lindberg et al. (2018) used density functional theory (DFT) calculations to predict the interaction energy between O_2_ and different electrolytes, among them DMSO. The geometry of the most stable complex for the [O_2_: DMSO] combination corresponds to the interaction of oxygen atoms of O_2_ with hydrogen atoms of methyl groups in DMSO. Belletti et al. (2019) demonstrated by DFT calculations that nine DMSO molecules are surrounding the O_2_ molecule in the first coordination sphere. Thus, it can be assumed that in the presence of O_2_ there is the formation of a [O2:DMSO] complex where solvent molecules stabilize a positive charge (from a fructose dehydration intermediate) more easily, favoring the mechanism of 5-HMF production. According to Kimura et al. (2013), this non-catalytic fructose conversion in DMSO allowed the production of 5-HMF after about 45 min of reaction at 90°C. Prior to this, fructose is converted into a primary precursor, which will undergo subsequent dehydration give 5-HMF.

With longer reaction times, similar fructose conversion values were obtained in the presence of O_2_, in the blank reaction and in the carbon catalyzed reaction (Figures 6, 7). However, reactions in the absence of heterogeneous catalyst showed lower selectivity to 5-HMF compared to values obtained in the carbon catalyzed reactions.

At the same temperature, the catalyzed reactions under synthetic air allowed a higher conversion of fructose. After 1 h of reaction under N_2_ the maximum conversion was 10.3% while in the presence of O_2_ fructose conversion reached 36.3%. The system under N_2_ remains less active at longer reaction times, but a smaller difference was observed. After 4 h reaction time a maximum fructose conversion of 77.8% was obtained under N_2_, while under synthetic air this value reached 83.3%. In contrast, the values obtained for the 5-HMF selectivity in reactions under N_2_ was higher than those obtained under synthetic air until 1 h reaction time, reaching a selectivity of 99.7% after 0.5 h at 100°C. After the first hour of reaction, the drop in 5-HMF selectivity is more pronounced under N_2_, but no significant increase in levulinic and formic acids production was observed.

**Figure 8:**
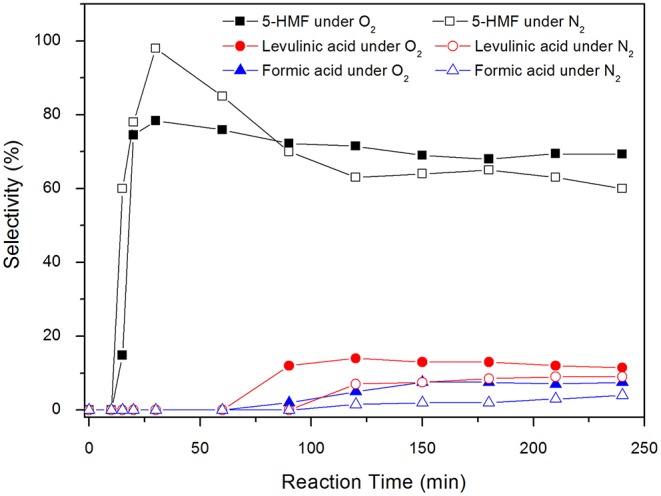
Selectivies toward 5-HMF, formic acid and levulinic acid for carbon catalyzed reactions under inert atmosphere and under synthetic air at 100°C. Conditions: 5% fructose in DMSO, 5 wt.% catalyst.

The selectivies toward 5-HMF, levulinic acid, and formic acid in the carbon catalyzed reactions are presented in Figure 8.

Levulinic acid is only observed after 90 min of reaction. It is worth noting that, in the presence of carbon, 5-HMF is produced from the beginning of the reaction (Figure 8), reaches a maximum after 30 min and then slightly decreases throughout the reaction. In the absence of carbon, the system takes about 30 min to produce 5-HMF and after 90 min of reaction the production of levulinic acid begins. Up to 30 min reaction time, the selectivity values of 5-HMF are increasing, reaching a maximum of 98%. In this initial stage of the reaction, part of the fructose must be in the form of intermediates that correspond to the dehydration steps necessary for the formation of 5-HMF. Although they were not quantified by HPLC, these intermediaries were already identified by different techniques (Zhang et al., 2016), where the authors found behavior similar to that shown in the Figure 7.

The selectivity values for the by-products levulinic and formic acids were higher in the blank reactions. 5-HMF was only identified after 1 h reaction time under N_2_, with a selectivity of 58%. This value remained practically unchanged throughout the reaction, reaching 56.5% after 4 h. Levulinic acid was observed only after 90 min, reaching a maximum selectivity of 23% after 2 h reaction time. After that, selectivity suffered a continuous reduction of up to 12% in 4 h. The behavior of formic acid was similar, with a maximum selectivity of 15% in 2 h and 10% after 4 h.

The yield of levulinic acid in the reaction carried out under synthetic air reached 9.1%, while in the reaction under inert atmosphere, this value was 7.0%. Similarly, formic acid yield reached 6.2% under synthetic air and 3.1% under N_2_. This result corroborates the assumption made by Whitaker et al. (2019), who suggested that the production of organic acids is enhanced in the presence of oxygen. The production of humins increases during the reaction, reaching a yield of 10.1% in the presence of O_2_ and 21.0% under inert atmosphere. Over time, a strong brown color, indicative of a higher concentration of humins, was observed in reactions under N_2_ in both temperatures, 130° and 100°C. The color intensity was higher than that observed in reactions made under synthetic air. The favoring of humins formation at higher temperatures is reported in the literature by Shahangi et al. (2017) who observed that as reactions were promoted at higher temperatures, there was a loss in selectivity of 5-HMF and the formation of an increasingly intense brown color, indicating the presence of humins.

Comparing the reactions under synthetic air or N_2_ at the same temperature, the higher formation of humins in inert atmosphere is related to the high amount of 5-HMF present in the former reaction period. Yalpani (1985) and Petronijevic et al. (2013) demonstrated several reactions in which DMSO shows crosslinking agent behavior with polysaccharides, when in the presence of aldehydes and organic acids. The presence of high concentrations of 5-HMF and fructose in the reaction medium must have enhanced the ability of DMSO to promote crosslinking, favoring fructose polymerization and humin formation. It is noteworthy that Tsilomelekis et al. (2016) demonstrated that levulinic acid is not significantly incorporated into humins, but aldolic condensation between 5-HMF and the ketone group must occur. Therefore, inhibition of DMSO catalytic activity under inert atmosphere reduced the formation of levulinic and formic acids, but there is still the formation of humins as a byproduct.

**Figure 9:**
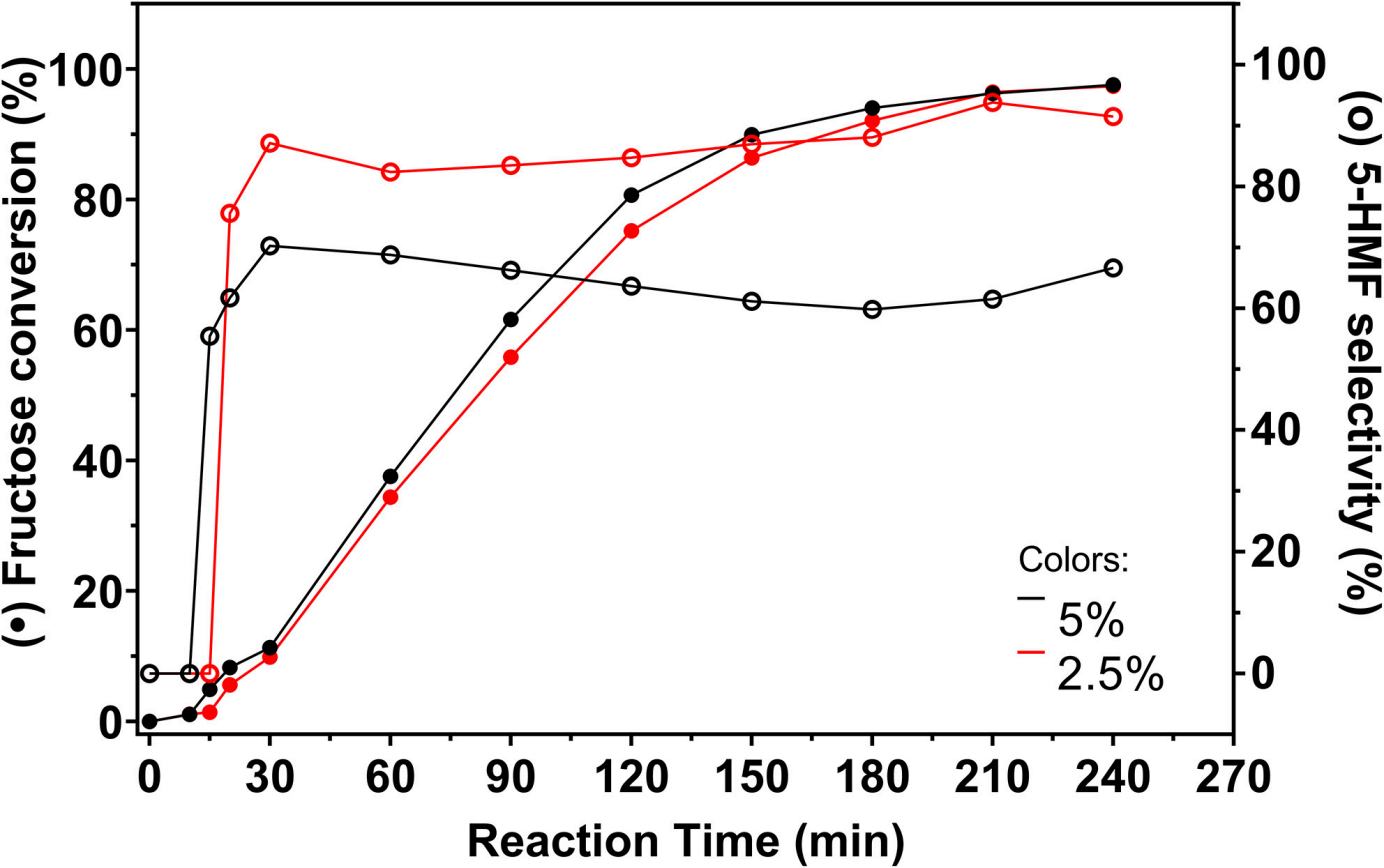
Fructose conversion and 5-HMF selectivity values (full and empty symbols, respectively) for carbon catalyzed reactions promoted from 2.5 and 5% fructose solutions in DMSO. Conditions: 130°C, inert atmosphere, 5 wt.% catalyst.

Since fructose and 5-HMF can polymerize, the fructose concentration in the system may be an important factor to consider in order to avoid the formation of humins. The influence of fructose concentration and catalyst amount is reported by Thapa et al. (2017). When the authors trying to produce levulinic acid from fructose, they noticed that the use of a higher fructose concentration and a lower catalyst amount caused a lower selectivity to levulinic acid and the consequent accumulation of fructose and 5-HMF led to a higher concentration of humins. Thus, solutions containing 2.5% fructose in DMSO were used to evaluate reaction behavior in the presence of lower fructose concentration. Fructose conversion and 5-HMF selectivity data are presented in Figure 9.

Fructose conversion reached similar values in both reactions, meaning that less fructose remained in solution when the reaction was conducted from a solution containing 2.5% fructose in DMSO. Selectivity to 5-HMF increased considerably with decreasing fructose concentration. This behavior is related to the minimization of polymerization reactions, since less fructose is available in the reaction medium. Much less intense staining was observed in reactions with 2.5% fructose, indicating that polymerization was better controlled.

**Figure 10:**
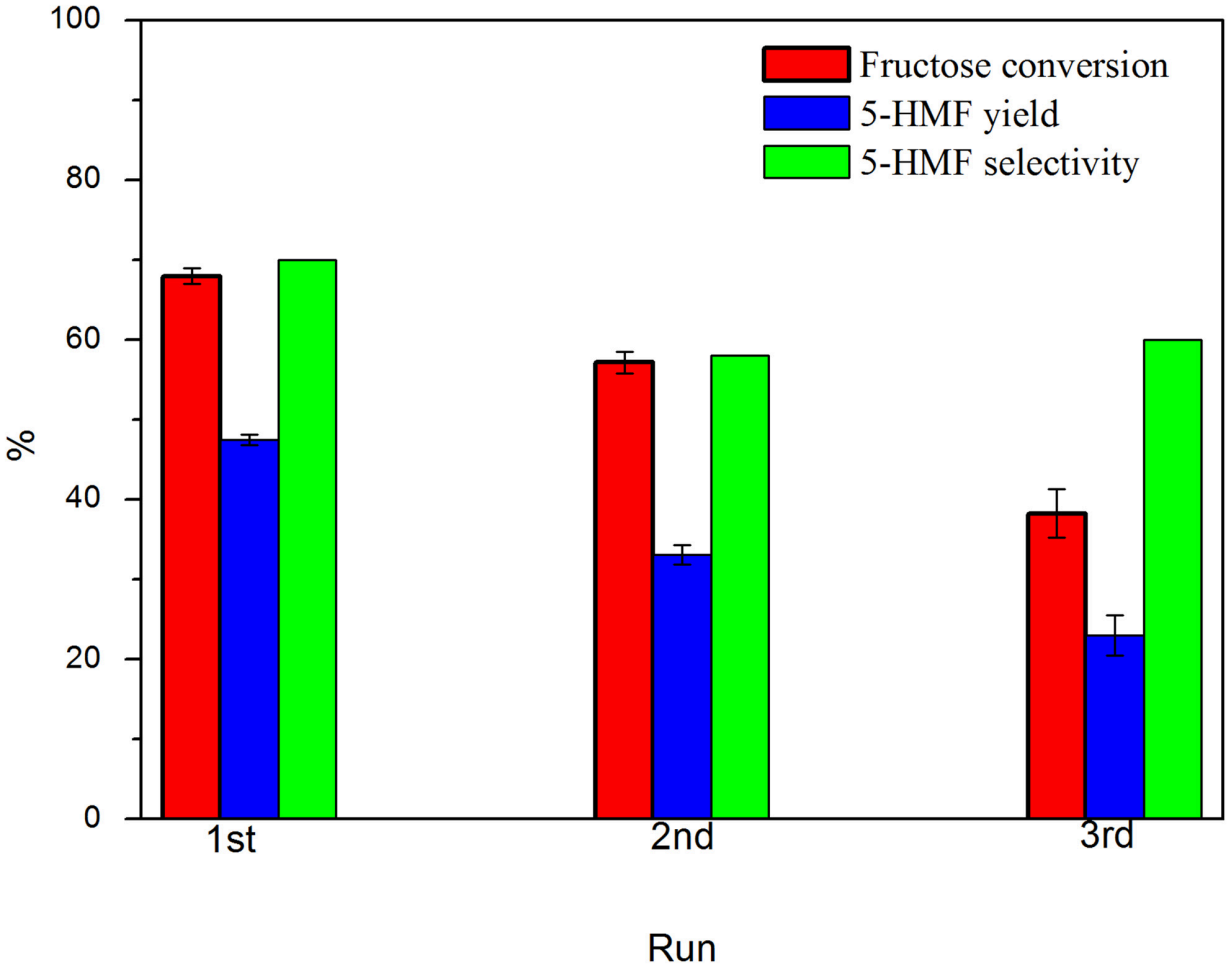
Catalyst reusability in fructose dehydration in DMSO. Conditions: 100°C, 5% fructose in DMSO, 5 wt.% catalyst, 2 h under synthetic air.

In order to investigate the reusability of the catalyst, consecutive batch runs were performed under the same reaction conditions. Results are presented in Figure 10.

It was observed a gradual lost in the catalyst performance after each run. From 1st to 2nd run, it was observed that the fructose conversion reduced from 68.5 to 57.2%, with a decrease of 12.5 percentual points in the 5-HMF selectivity. From 2nd to 3rd run, conversion reduced from 57.2 to 38.3%, while the 5-HMF selectivity has stabilized. This behavior is similar to that previously observed (Nunes et al., 2020) and also reported in other works (Portillo Perez et al., 2019; Rusanen et al., 2019). The main reasons found in the literature are (I) loss of -COOH/-SO3H groups and (II) deactivation of the acid sites by deposition/anchoring of humins on carbon surface. Some groups from the solid surface can leach to the reaction medium under the reaction conditions, thus leading to homogeneous catalysis. In order to investigate the surface groups leaching, a reaction was performed, wherein the catalyst was removed from the reaction medium after 30 min of reaction, keeping the system temperature at 100°C, as proposed by Sheldon et al. (Sheldon et al., 1998). Fructose conversion value observed after 30 min remained for 3 h after catalyst removal. This means that the homogenous contribution, arising from the leached soluble groups, can be considered negligible. On the other hand, several authors have observed that a minimal amount of humins can block the acidic sites of the catalyst, leading to its gradually deactivation (Portillo Perez et al., 2019; Nunes et al., 2020). A XPS analysis on the spent catalyst provided the composition 78.8% C, 20.2% O, and 1.0% S. When comparing the result with the values obtained previously, it is noted that a slight increase in the oxygen content, while the amount of sulfur on the surface has been drastically reduced. The results corroborate the hypothesis of the humins deposit on the catalyst surface, blocking the original acid groups. In some cases, a washing process with acetone was able to remove the humins and recover the activity of the catalyst. In conditions similar to this work, it was observed that the removal of humins by a solvent washing is not efficient enough for the reactivation of the catalyst and, in this case, a recarbonization process allows the maintenance of the catalyst activity, suggesting that humins on the spent catalyst were carbonized and incorporated on carbon surface, creating a new layer of acidic sites.

## Conclusions

The catalyst based on *in situ* carbonization and sulfonation allowed to achieve fructose conversions close to 100% with 5-HMF selectivities higher than 90%. This catalyst can be used in consecutive batch runs, but it was observed a gradual lost in the catalyst performance after each run due to deposition of humins on the carbon surface. Maintaining the activity requires recarbonization of the solid.

Proper control of DMSO catalytic activity can be achieved in an inert atmosphere. The presence of oxygen in the reaction medium accelerates the occurrence of fructose dehydration reaction, which may provide a higher production of 5-HMF, but also favors the decomposition of this product, with higher formation of levulinic and formic acids. In reactions under inert atmosphere the occurrence of rehydration reactions can be better controlled. Therefore, DMSO activity, in the presence of oxygen and at high temperatures, was considered as the major influence for the occurrence of rehydration and polymerization reactions parallel to the formation of 5-HMF.

Decreasing the initial concentration of fructose and the catalyst content in the reaction medium minimized polymerization reactions, enabling higher selectivity to 5-HMF even under high temperature conditions where polymerization is favored.

Despite the catalytic capacity of DMSO in the presence of O_2_, the use of a glycerol carbon as catalyst favored fructose conversion and 5-HMF production. It is noteworthy that several studies in the literature do not consider the blank reaction and, consequently, the contribution of DMSO to the conversion of fructose, especially at temperatures above 80°C and in the presence of oxygen, mistakenly attributing all activity to the heterogeneous catalyst.

The results indicated the appropriate conditions for using DMSO, contributing to the search for green solvents, since there is a compromise between effectiveness of the DMSO application as a solvent and its relative abundance, low cost and reduced toxicity when compared to competing solvents.

## Data Availability Statement

All datasets generated for this study are included in the article/Supplementary-Material.

## Author Contributions

WC and DM contributed conception and design of the study, TT performed the synthesis and analysis, TT and RN wrote the first draft of the manuscript, WC wrote the final version of the manuscript. All authors contributed to manuscript revision, read, and approved the submitted version.

### Conflict of Interest

The authors declare that the research was conducted in the absence of any personal, professional or financial relationships that could potentially be construed as a conflict of interest.
